# Correction: Highly efficient production of transgenic rats with long DNA insertions using *piggyBac* transposase mRNA and piezo-assisted microinjection

**DOI:** 10.1371/journal.pone.0345469

**Published:** 2026-03-19

**Authors:** The PLOS One Staff

In the Abstract section, there is an error in the fourth sentence. The correct sentence is: The use of *piggyBac* transposase have also been examined in rats.

In the Abstract section, there is an error in the ninth sentence. The correct sentence is: We also attempted to generate a rat model of Alzheimer’s using this method.

In the Introduction section, there is an error in the sixth sentence. The correct sentence is: Even when the survival rate of zygotes is high, the birth rate of the rats is not particularly high (16.6%–23.0% [1]; 3.7%–32.3% [2]; 25.1%–30% [3]; 31.7% [4]; 16.7%–38.7% [5]; 3%–28% [6]) and the proportion of Tg rats among the offspring remains low (6.3%–15.2% [1]; 4.3%–33% [2]; 12.1%–13.6% [3]; 18.5% [4] 5.7%–16.7% [5]; 0%–41% [6]; 0.7%–5% [7]).

The publisher apologizes for the error.
