# Supplementary figures and images for: Highly efficient production of transgenic rats with long DNA insertions using piggyBac transposase mRNA and piezo-assisted microinjection
Source: PLoS One. 2026 Feb 17;21(2):e0339406. doi: 10.1371/journal.pone.0339406 (PMC12912583; doi:10.1371/journal.pone.0339406)

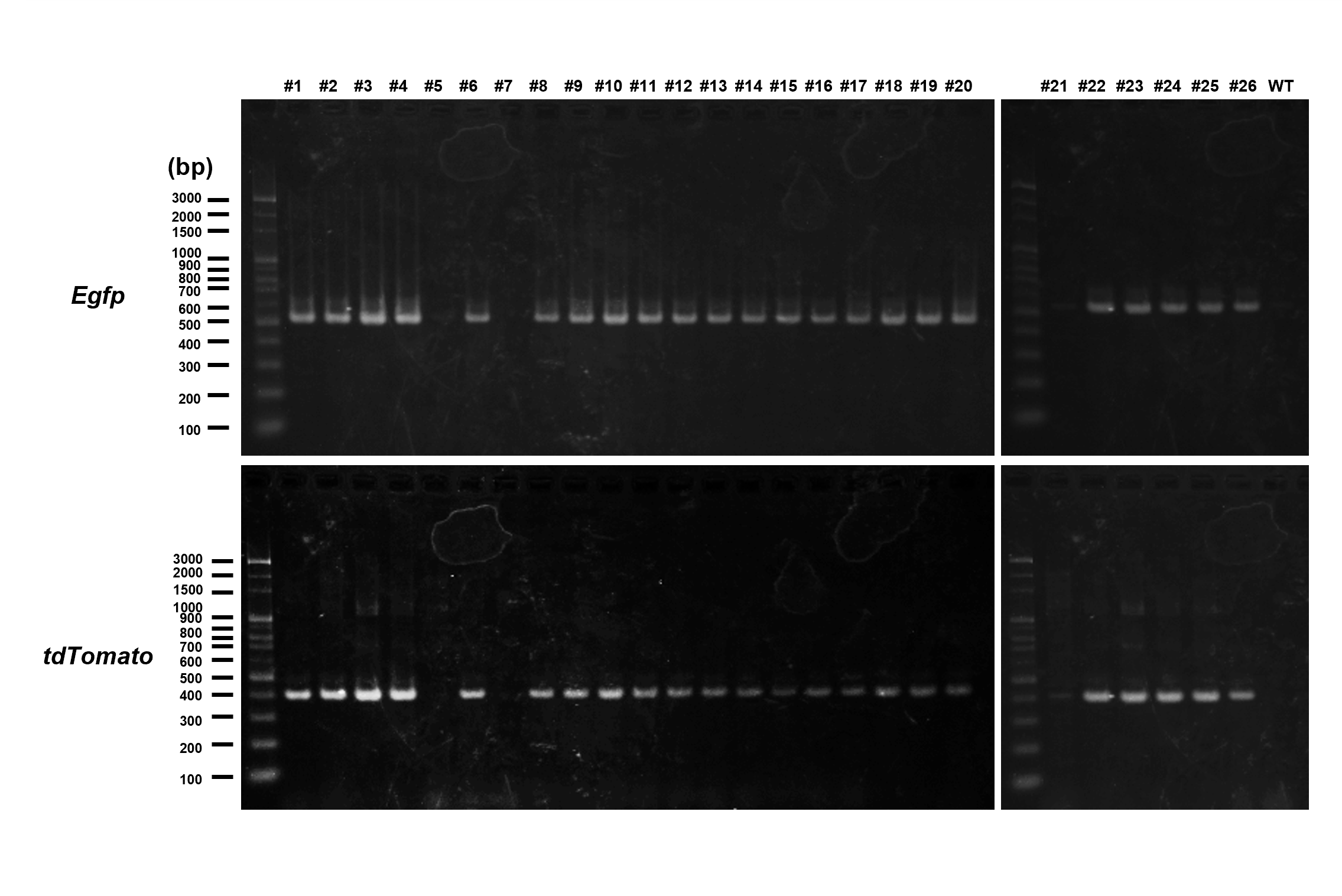

Supplement: S1 Fig — Images of uncropped and minimally adjusted agarose gels corresponding to Fig 1C. (TIF) [file pone.0339406.s001.tif]

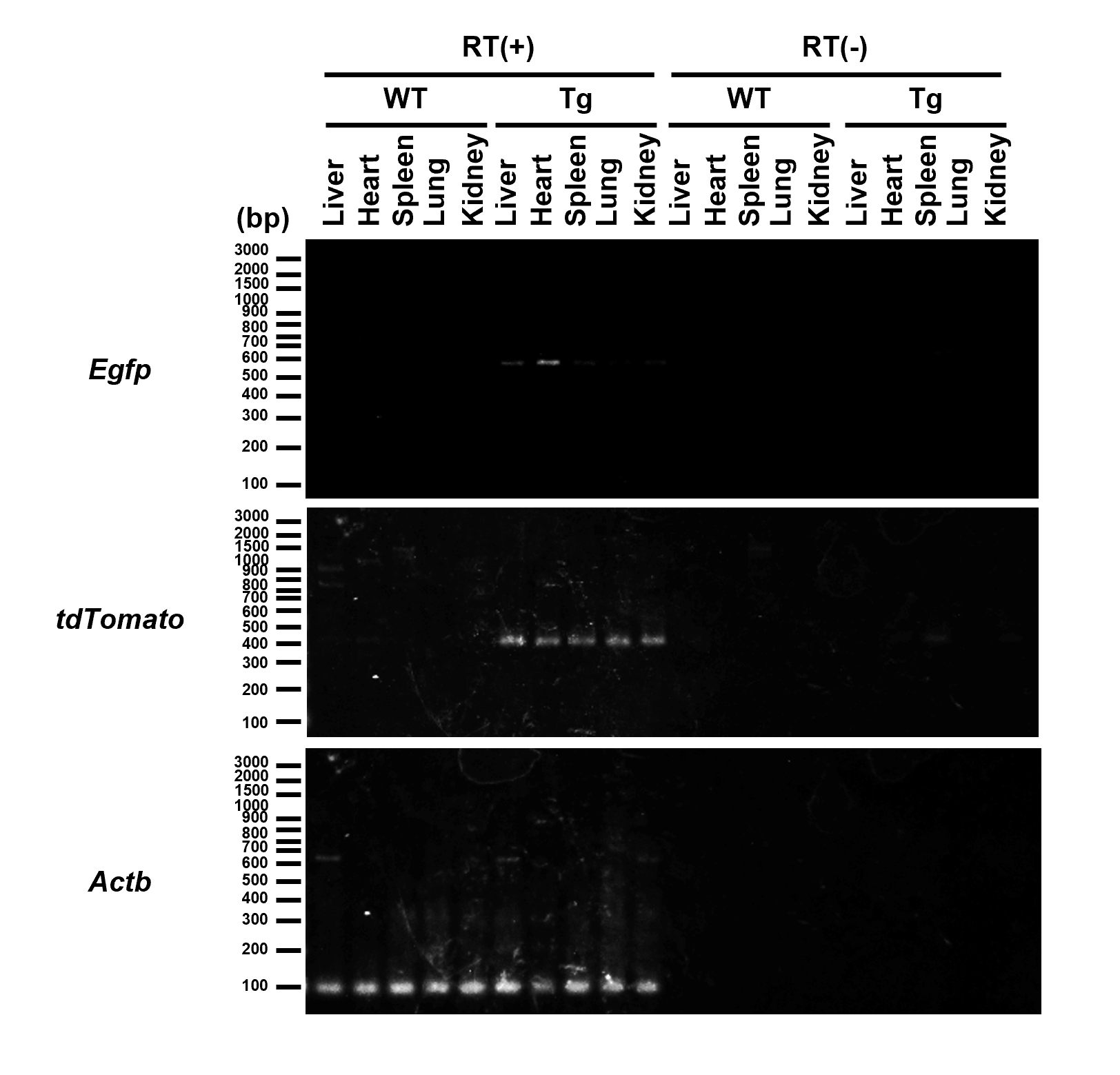

Supplement: S2 Fig — Images of uncropped and minimally adjusted agarose gels corresponding to Fig 2A. (TIF) [file pone.0339406.s002.tif]

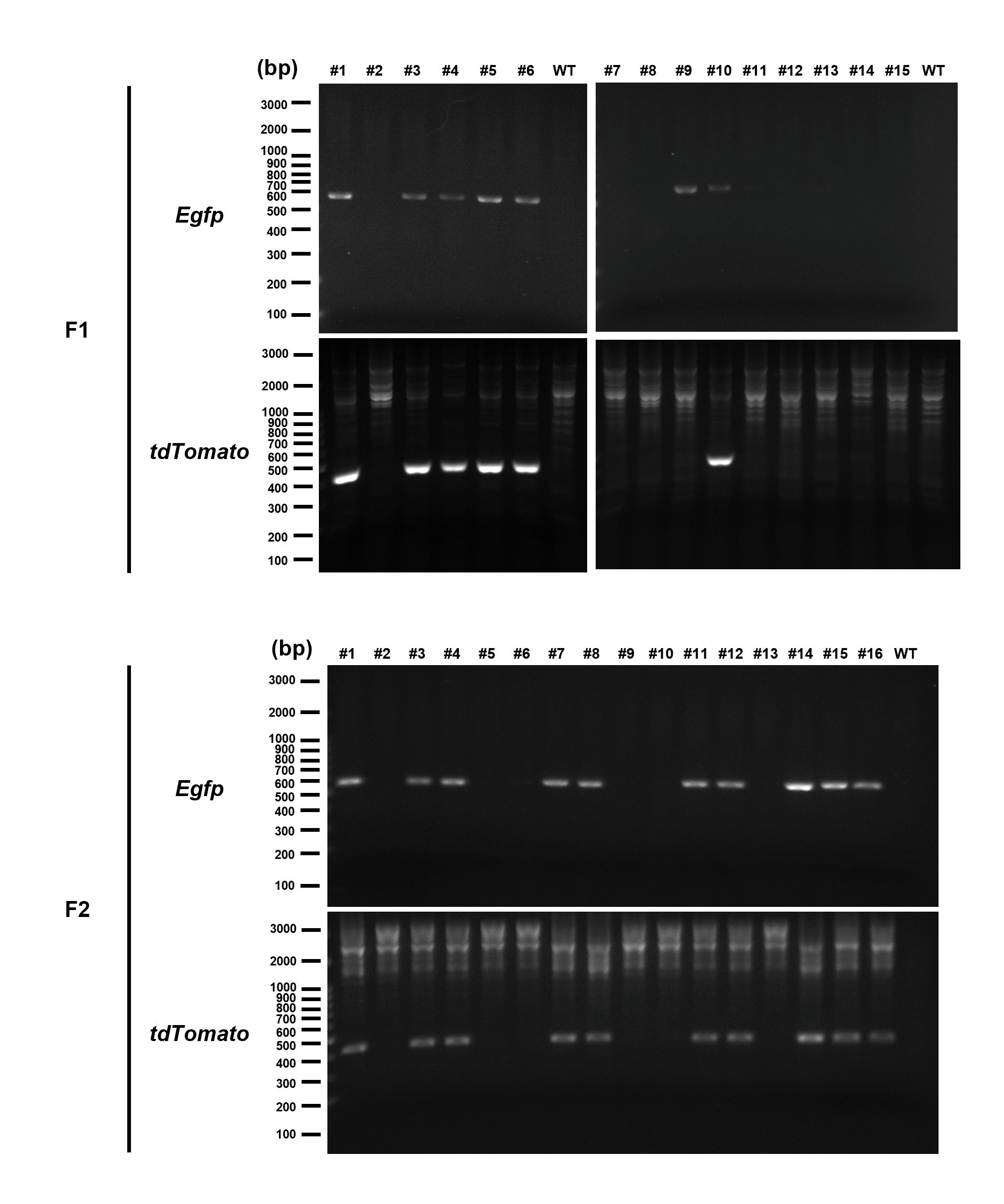

Supplement: S3 Fig — Images of uncropped and minimally adjusted agarose gels corresponding to Fig 3B and F. (TIF) [file pone.0339406.s003.tif]

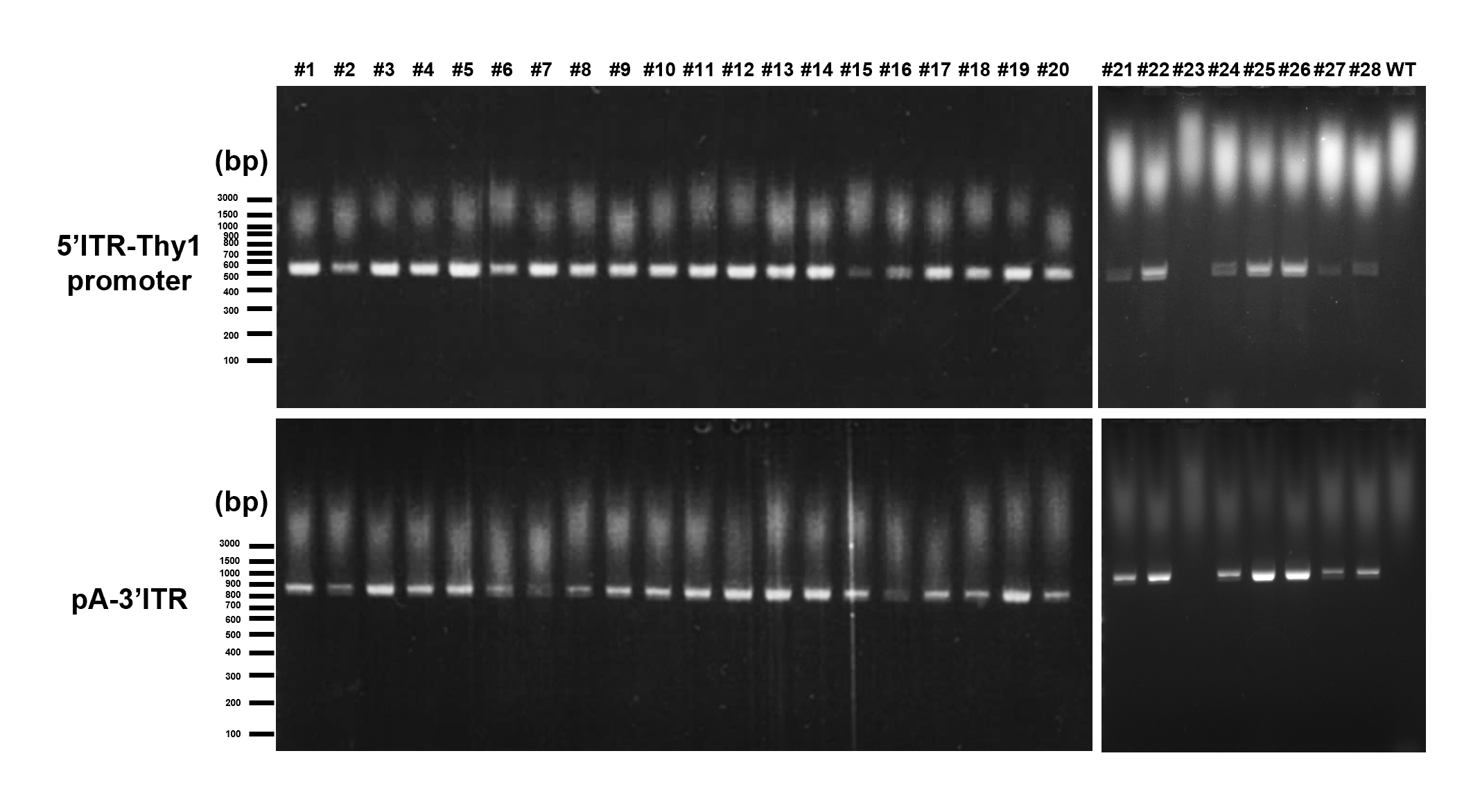

Supplement: S4 Fig — Images of uncropped and minimally adjusted agarose gels corresponding to Fig 4B. (TIF) [file pone.0339406.s004.tif]

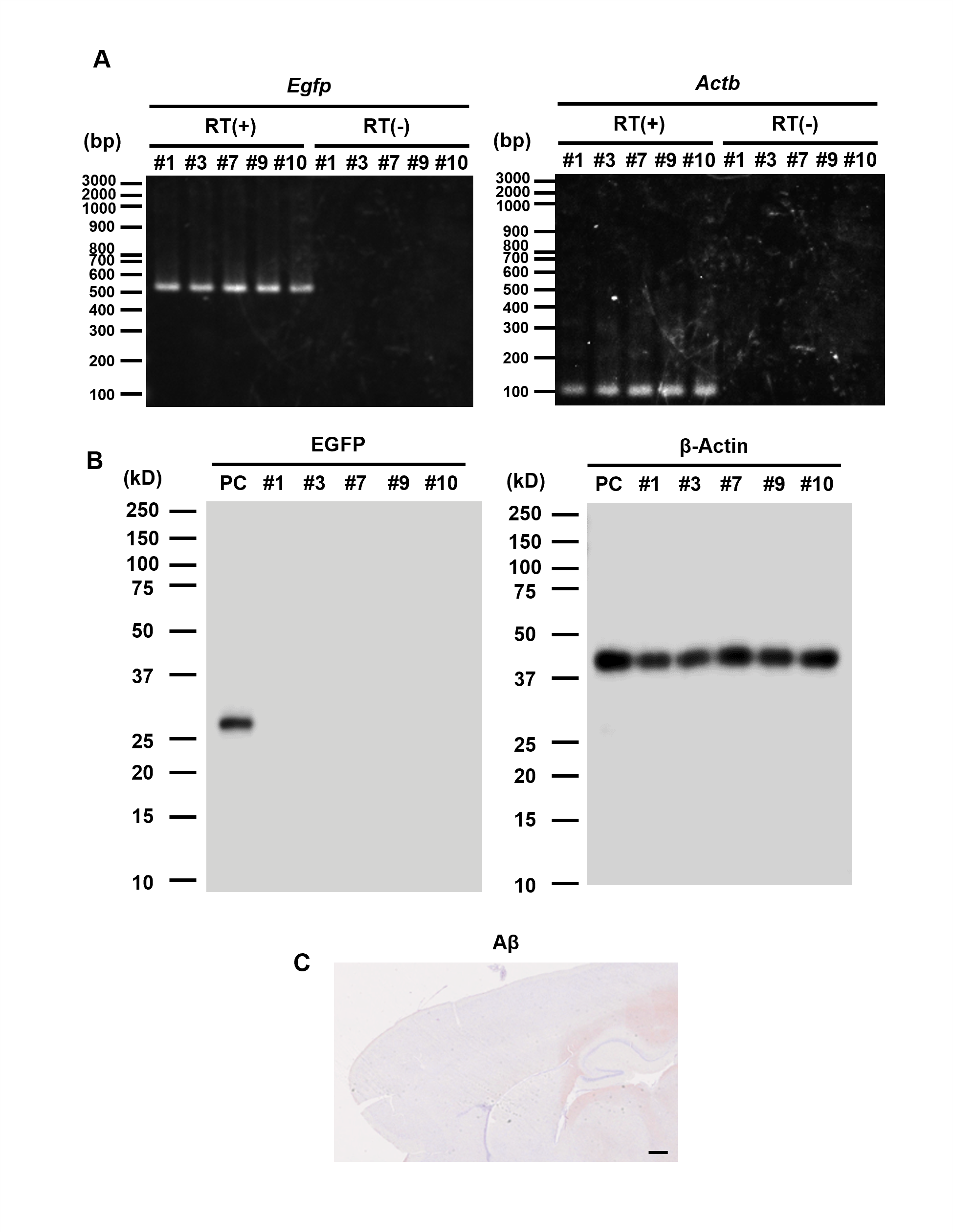

Supplement: S5 Fig — (A) Analysis of transgene mRNA expression in cerebral cortex by RT-PCR. Images of uncropped and minimally adjusted agarose gels. (B) Analysis of transgene protein expression in cerebral cortex by western blot. PC: positive control (CAG-EGFP Tg rat). Images of uncropped and minimally adjusted blots. (C) Immunohistochemistry of Aβ in cerebral cortex. Scale bar: 1 mm. (TIF) [file pone.0339406.s005.tif]

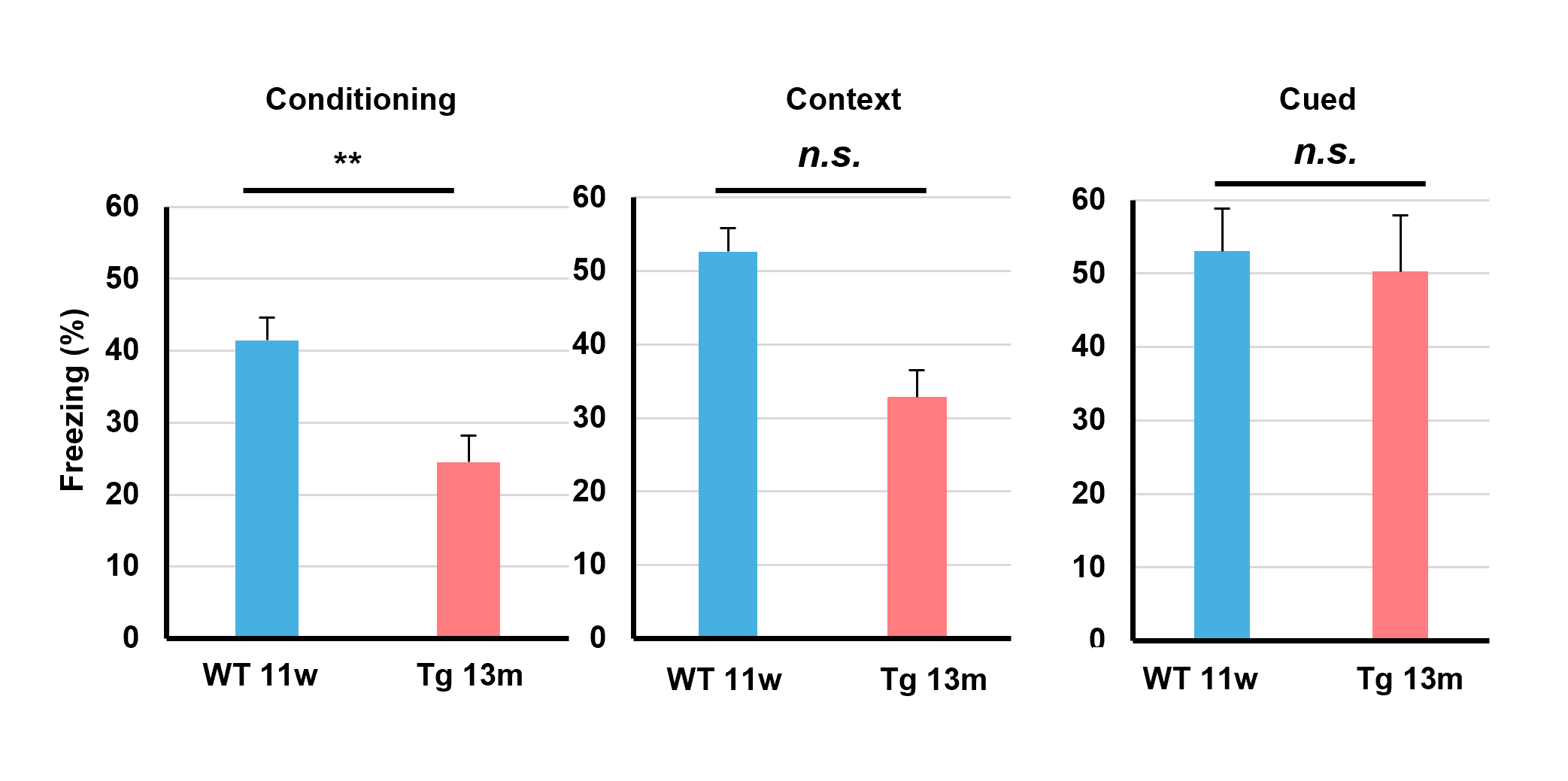

Supplement: S6 Fig — 5xFAD Tg rats at 13-month-old and WT rats at 11-week-old were used. *p < 0.05, **p < 0.01, n.s.: not significant. (TIF) [file pone.0339406.s006.tif]
