# Supplementary material for: Highly efficient production of transgenic rats with long DNA insertions using piggyBac transposase mRNA and piezo-assisted microinjection
Source: PLoS One. 2026 Feb 17;21(2):e0339406. doi: 10.1371/journal.pone.0339406 (PMC12912583; doi:10.1371/journal.pone.0339406)
Supplement: Attachment — Submitted filename: Response to reviewer comments for revision.docx [file pone.0339406.s013.docx]

Response to reviewer comments:

We sincerely thank the reviewer#1 for their careful evaluation and constructive comments, which have helped us improve the clarity and scientific quality of our manuscript.

Overall Assessment

*This manuscript presents a study describing a highly efficient method for generating transgenic rats using piggyBac transposase mRNA and donor plasmid DNA combined with piezo-assisted pronuclear microinjection. The authors demonstrate high zygote survival rates in comination with a high transgenic efficiency, even when using large donor DNA constructs (>10 kb). The method is clearly described and experimentally validated across F1 and F2 generations.*

*The study is technically sound, addresses a real bottleneck in rat transgenesis. Although it should not be understated that the technique still requires a high level of technical skill in microinjection and familiarity with piezo-assisted systems. While the method may lower the barrier for experienced embryologists by improving reproducibility and zygote survival, it does not entirely eliminate the need for precise micromanipulation expertise.*

Response

We appreciate the reviewer’s positive overall assessment and insightful comments. We fully agree that the current method still requires technical proficiency in microinjection and familiarity with piezo-assisted systems. We have included an additional note in the Discussion section clarifying that limitations remain, including the need for micromanipulation skills and the requirement for piezo equipment (Line 339—341). Although future development of simpler approaches such as electroporation-based delivery would be desirable, these methods are not yet as efficient or reliable in mammals. Therefore, we believe that the present method currently offers the most effective strategy for generating transgenic rats with high efficiency and reproducibility.

2. Validity of the Methods

• *The experimental design is clear and reproducible, with detailed descriptions of plasmid preparation, microinjection, embryo transfer, and genotyping.*

*• Ethical approval and animal welfare considerations are properly stated.*

*• The use of droplet digital PCR (ddPCR) for copy-number quantification is appropriate and provides solid validation.*

Response

We thank the reviewer for the positive evaluation of our experimental design and methodology. We also thank the reviewer for acknowledging our attention to ethical and animal welfare considerations, as well as the appropriate use of ddPCR for accurate copy-number quantification.

*In the Materials and Methods section, please add which strain was used for embryodonation and which one for the embryo transfer etc. (Line 70).*

Response

We acknowledge the reviewer’s helpful suggestion and have added information specifying the strains used for embryo donation and embryo transfer in the Materials and Methods section (Line 71—74).

*In addition, include the anesthesia protocol including pain management, as the cited source is not publicly accessible (line 113).*

Response

We thank the reviewer for this valuable suggestion. The editor made a similar comment, and I have added the composition of a mixture of three anesthetic agents to the protocol in line 75—78.

3. Soundness of the Results and Data Interpretation

*• The high integration rate and germline transmission are convincing and strongly support the authors’ claims.*

*• The observed correlation between high copy number and postnatal lethality is interesting and consistent with known effects of overexpression from strong promoters.*

• *The 5xFAD rat model section is less successful: despite mRNA expression, the lack of protein and phenotype suggests translational inefficiency or construct design issues (e.g., promoter incompatibility or 5′-UTR sequence effects). This section could be condensed or moved to the Supplementary Information, as it distracts from the main technical findings.*

Response

We are grateful for these positive and insightful comments. We agree that the high integration rate, germline transmission, and the observed correlation between high copy number and postnatal lethality support the validity of our findings and are consistent with previous reports on promoter-driven overexpression effects. We also appreciate the valuable feedback regarding the 5xFAD Tg rat generation section. Although we could not achieve successful establishment of Alzheimer's disease model by generating the 5xFAD Tg rat line, we have retained the data up to the efficient generation of recombinant embryos in Fig. 4. According to the reviewer’s suggestion, we have moved the expression and behavioral analyses (Fig. 4D—H) to the Supplementary Information (S Fig. 5 and 6). In addition, we have moved part of the description of the 5xFAD Tg mice originally included in the Results section to the Discussion section (Line 327—330).

4. Appropriateness of the Conclusions

*The conclusions are well supported by the data. The authors correctly state that the piggyBac mRNA plus piezo-injection method allows for efficient production of transgenic rats with long DNA inserts.*

*However statements about the simplicity and broad applicability of the method should be tempered with acknowledgement of limitations (e.g., requirement for piezo equipment, variable copy numbers and possible embryonic toxicity).*

Response

We thank the reviewer for the constructive comment. The conclusion has been revised to acknowledge the limitations of the method, including the requirement for piezo equipment, the need for basic skill in micromanipulation, and the possibility of embryonic toxicity depending on the transgene copy number (Line 342—344).

5. Clarity and Presentation

*The manuscript is generally well written and logically structured.*

Response

We are pleased that the reviewer found the manuscript clear and logically structured. Minor edits were made to further improve readability.

6. Recommendation

Decision: With minor revisions, this manuscript is suitable for publication in PLOS ONE.

Response

We are grateful to the reviewer for recommending our manuscript for publication after minor revision. We have carefully considered all comments and made corresponding revisions to improve the manuscript.
